# Sexual (Dys)function after Urethroplasty

**DOI:** 10.1155/2016/9671297

**Published:** 2016-03-09

**Authors:** Luís Xambre

**Affiliations:** Centro Hospitalar de V. N.Gaia/Espinho, Serviço de Urologia, Rua Conceição Fernandes, 4434-502 Vila Nova de Gaia, Portugal

## Abstract

There is a paucity of published literature on the andrological consequences of urethral repair. Until recently authors have focused mainly on technical aspects and objective results. Reported outcomes of urethral reconstruction surgery have traditionally focused only on urodynamic parameters such as flow rates. Patient reported outcome measures have largely been neglected and there is a scarcity of well conducted systematic studies on the subject. For these reasons whether the different components of sexual life are more or less affected by different types of urethral reconstruction remains largely unknown. In an attempt to clarify the available scientific evidence, the authors make a critical review of available literature, systematizing it by sexual domain and study type. Brief pathophysiological correlations are discussed.

## 1. Introduction

Urethral stenosis, although relatively uncommon in the universe of urologic diseases, is by no means a rare condition. It accounts for about 52% of urethral and 1.8% of urologic pathology, respectively, and presents an estimated prevalence of 0.6% [1, 2]. Relatively young, active individuals are mostly affected. Its association with an unequivocal negative impact on the quality of life, whether resulting from the disease itself and its complications or whether consequence of the treatment(s) employed, is well established.

At present, there is no doubt that reconstructive surgery in the form of different types of urethroplasty represents the “gold standard” in the treatment of these patients. Urethroplasty is associated with reproductively high success rates, when properly employed. There is enough data in the literature regarding the results obtained with several techniques, anastomotic or substitution. When objective variables such as flow rates are considered, several authors describe success rates that exceed in many cases 80% whether for anterior urethra, bulbar [3–5] or penile [6–8], or whether for posterior urethra [9–11].

These data reflect however only one aspect of results, as patients carry out a substantially different perception of success than physicians, not only taking into account flow rates and radiological or endoscopic data. It is well known that there is a significant mismatch between what is considered a urethral reconstruction failure/success between treatment physicians and patients [12–14]. Aspects such as aesthetics or those related to sexual function are obviously important from the perspective of patients and often overlooked in the literature. If we do a simple exercise as, for example, an electronic search using the most widespread scientific literature database, PubMed, this disparity becomes obvious. If researches using terms like “urethroplasty and results” provide thousands of references, terms such as “urethral stenosis” or “urethroplasty” and “erectile dysfunction” or “impotence” result in only a few dozen scientific papers. Moreover, many books that specifically address urethral reconstruction almost exclusively focus on anatomical or technical aspects and there is a virtual absence of information about the andrological aspects of urethroplasty.

Although in recent years there has been a growing interest in relation to urethral stricture's andrologic implications, the relationship between urethroplasty and erectile dysfunction, for example, remains controversial up to the present day. The existence of few specific studies, heterogeneous study populations, differing methodologies, and diversity of procedures analyzed makes it very difficult to provide definitive answers.

*Pathophysiology of Sexual Dysfunction*. Surgical approaches involving the external genitalia have an unmistakable noxious potential in several domains of sexual function, aesthetic and dysmorphic changes.

Consequences in aesthetics and change of body image, mostly related to the distal urethroplasties, have obvious potential impact in terms of self-esteem and possibly sexual behavior. Although of subjective nature, these aspects are particularly noticeable in multioperated hypospadias patients, a group of patients of increasing importance in percentage terms that pose a particularly difficult approach.

Concerning erectile and ejaculatory dysfunction, potentially injured structures in the course of urethroplasty include several arterial structures, nerve branches (autonomic and/or somatic), and eventually myogenic components.

There is a recognized potential for injury of branches of the Common Penile Artery, essential in the hemodynamics of erection in posterior urethroplasties, and of more distal vessels, of smaller and questionable practical importance, in anterior urethroplasties.

Equally important are neurogenic autonomic lesions due to the proximity of the neurovascular bundles to the membranous urethra, potentially damaged in instrumentation of the posterior urethra [15, 16]. Somatic neurogenic components, either sensory or motor, involving the dorsal penile or perineal nerve and its branches, are also at risk, particularly during anterior urethroplasties [17–19].

Of potential functional importance, though debatable and still practically in the field of scientific curiosity, are the neuronal connections identified between autonomic and somatic pelvic, perineal, and even genital nerve terminals, making the latter capable of nitrergic activity. Authors like Yucel and Baskin [20] and Alsaid et al. [21] using immunohistochemistry-based studies in fetuses unequivocally demonstrate connections between the neurovascular bundles from the pelvic plexus, nitrergic, and components of the somatic nervous system (branches of the pudendal nerve such as the dorsal nerve of the penis and perineal nerve), giving them the capacity to release erectogenic mediators.

Finally, section and aggressive mobilization or denervation of the bulbospongiosus muscle to expose the bulbar urethra may result in more or less subtle changes in ejaculation dynamics, since the rhythmic contractions of the muscle during the expulsion phase are fundamental in seminal fluid expulsion [22–24].

## 2. Materials and Methods

A systematic review of several databases including PubMed, Cochrane Library, Embase, and Google Scholar was conducted. Systematic searches of these databases used terms as “urethroplasty,” “urethral reconstruction,” “urethral anastomosis,” “urethral stricture,” “urethral stenosis,” and “urethral obstruction,” and terms such as “erectile dysfunction,” “impotence,” “sexual dysfunction,” “ejaculatory dysfunction,” and “orgasmic dysfunction.” The search strategy used both keywords and MeSH terms and was limited to human studies.

The purpose of this study was to review the existing literature about the impact of urethroplasty in all domains of sexual function and to analyze it.

## 3. Results and Discussion

### 3.1. Body Image/Self-Esteem

Literature is absolutely lacking in terms of evaluation of the aesthetic consequences of urethroplasties performed in adulthood for urethral strictures. We can only infer conclusions based on findings from literature in the context of hypospadiology, a study population with necessarily different and very particular characteristics.

Despite all the limitations pointed out, there are a few studies that looked specifically at the cosmetic aspect of the reconstruction of the penile urethra in this context that allow us at least some critical reflections.

Authors as Bubanj et al. [25] used a postal questionnaire including questions about genitosexual functioning and sexual behavior in a comparative study of 37 patients submitted to urethroplasty for hypospadias repair 2–15 years earlier (mean age 27.8 ± 6.2 years/average number of surgeries 3.81 ± 3.37) and a group of 39 normal men (mean age 25.5 ± 5.3 years). No significant differences were found between the groups with regard to inhibition of search for sexual contacts or sexual relationship patterns. Participants in both groups were mostly satisfied with their body image (83,78% of patients with hypospadias versus 89.74% in the control arm). However, there were significant differences between the groups regarding the frequency of sexual activity and number of sexual partners. Only 51.35% of men with hypospadias regarded their sex life as fully satisfying against 76.92% of the control group.

Even et al. [26] analyzed a group of 15 young adult hypospadias patients (mean age 21.2 years) operated in childhood, employing instruments such as EuroQol 5, IIEF-15, and a nonvalidated questionnaire. One-third of patients thought that overall quality of life was distorted, although 80% were mostly satisfied with their sexual quality of life. The most important complaints were relative to the penile appearance.

Although subject to wide variation in individual perception, these aspects must of course be considered in addressing these patients and integrated with the other facets of the pathology/treatment strategy.

### 3.2. Erectile Dysfunction

#### 3.2.1. Anterior Urethra: Prospective Studies

There are few prospective studies with correct methodology, making use of fully validated questionnaires such as the IIEF (International Index of Erectile Function) or the BMSFI (Brief Male Sexual Function Inventory) specifically dealing with anterior urethroplasties.  Table 1 [27–31] lists these studies and the results obtained. It is evident that all these studies show statistical limitations. The small sample size by itself can obscure statistically significant differences simply as a consequence of underpowered studies. In fact, none of the studies makes explicit reference to calculations in order to define a minimum sample size that would be required to show 5 to 10% difference in outcomes for example. Although with relatively small samples and relatively short follow-up, the overwhelming majority of studies did not find statistically significant degradation of erectile function after urethroplasty compared to baseline.

The only discordant study is from Erickson et al. [31]. These authors analyze prospectively 52 men with penile or bulbar urethral stenosis subjected to several types of urethroplasties (penile urethra: ventral onlay or inlay, 2-stage; bulbar urethra: end-to-end anastomotic or augmented anastomotic repair). Patients were evaluated serially every 3 to 6 months by the IIEF.* De novo* erectile dysfunction (ED) was defined as an IIEF decrease of at least 5 points and recovery as a score less than 2 points from baseline. Although ED was observed in 38% of patients, 90% of these were found to recover function in accordance with established criteria, with an average recovery time of 190 days.

#### 3.2.2. Anterior Urethra: Retrospective Studies

Table 2 [32–38] summarizes the available retrospective studies that used structured, validated, or nonvalidated questionnaires. Being retrospective, these studies already have inherent limitations in terms of methodology. Additional obvious limitations in terms of sample size in most of them and the frequent use of nonvalidated questionnaires add additional limitations in terms of produced scientific evidence. Moreover, unfortunately these are recurrent limitations in the reconstructive urethral surgery scientific literature.

Other studies [39–41] are available, although with debatable statistical quality, making it difficult to draw any valid conclusions. Data reported are mainly descriptive and the presence of preoperative ED was not uniformly reported. Often the presence of postoperative ED was asserted only by means of a simple question [39–41]. These series have focused above all on technical aspects and success rates, and ED was only briefly referred to. ED rates ranged from 0 [40] to 7% [41].

Concerning the studies included in Table 2, it is apparent that there is some disparity regarding the employed methodology and the way results are presented. Authors like Singh et al. [32] and Erickson et al. [33] present a methodology perfectly intelligible and easy to read. These authors evaluated a cohort of patients submitted to different types of anterior urethroplasties using the same questionnaire. In both cases no statistically significant differences were found concerning the 3 specific questions related to erectile function of the BMFSI. For its part, Coursey et al. [34], perhaps in one of the first published papers specifically on the subject, report a 30.9% rate of degradation of erections after a mean follow-up of 36 months. It is interesting however that the authors used as a control group patients undergoing circumcision in which they found reduction in erectile performance in 27.3%, with no significant differences between the two groups. On the other hand, Morey and Kizer [36] Welk and Kodama [37] and Ekerhult et al. [38] are mainly series in which two techniques or technical variants (end-to-end, extended anastomotic, or onlay graft urethroplasty) are analyzed. There was no difference in the ED occurrence among the techniques. In all groups, reported ED rates were small.

#### 3.2.3. Other Prospective Studies: Anterior and Posterior Urethra

There are 3 additional prospective studies, presented in Table 3, involving mixed cohorts of patients undergoing anterior and posterior urethroplasties [42–44]. All used the IIEF-5 as assessment method. With follow-ups in the range of 6 to 27 months, no significant differences were found between pre- and postoperative scores by any of the authors.

#### 3.2.4. Posterior Urethra

Lesions of the posterior urethra, associated with the overwhelming majority of cases to traumatic injuries of the pelvic ring, are unequivocally linked to erectile dysfunction, either by direct damage to neurovascular structures or by indirect action of edema, inflammation, and fibrosis. Presence of urethral trauma in pelvic fractures is a widely documented risk factor of erectile dysfunction. 42% of patients with pelvic fracture and urethral lesions had ED compared with only 5% of patients with fractures and without urethral injury [45]. The literature accounts for ED percentages ranging from 18% or less to 72% [46], although the relative roles of the traumatic event and potential iatrogeny induced by reconstructive surgery remain unclear. Aspects such as lack of consensus on the definition of ED, heterogeneous series regarding the severity of the trauma, and obvious discrepancies in the evaluation methods explain the variability of results. The potential of spontaneous recovery up to about 24 months after the traumatic event is also widely documented, probably related to neuropraxia recovery and development of accessory vessels after vascular trauma. For this reason, the timing of the evaluation in relation to trauma and surgery plays an important role in result variability, since studies that report ED with evaluation 3–15 months after trauma present rates of ED significantly elevated (60–72%), when compared with series in which this evaluation was made at least 2 years after the traumatic event (18–32%) [46].

Table 4 is intended to summarize the existing literature on the subject [9, 10, 47–57]. It is quite apparent that the overwhelming majority of the series did not make use of validated questionnaires in the evaluation of patients and the evaluation methodology in relation to erectile function is, at most, only briefly mentioned. If trauma plays an obvious role in the etiology of ED, it is much harder to distinguish the specific role played by reconstructive surgery. Many of the studies only report global rates of ED after reconstruction and, as such, potentially encompass the effects of trauma and surgical iatrogenesis, more or less ameliorated by the aforementioned spontaneous recovery. Studies that specifically evaluate erectile function before and after urethroplasty are thereby especially enlightening.

Koraitim [10] analyzed a series of 155 patients who suffered posterior urethral trauma, although, without using any validated tool and not specifying the definition of ED used, the author refers to the fact that, of previously potent patients before trauma, 40% became impotent. Of the 66 patients without ED before urethroplasty only 2 were impotent as a result of surgery, in both cases after surgeries of great technical complexity, exceeding 9 hours. On the other hand, 29 of 44 previously impotent patients recovered erectile function after urethroplasty.

Analyzing 76 patients with a follow-up ranging from 14 to 74 months, Yin et al. [50] report an ED rate of 42% after trauma. Of the 58% of potent patients, 95% remained potent after urethroplasty and 5% developed* de novo* ED. 59% of impotent patients after trauma recovered erectile function after surgery.

Tunç et al. [54] described a series of 77 patients with posterior urethra injury treated with deferred urethroplasty after suprapubic diversion. 25.8% of the evaluated patients developed ED after trauma. Erectile function was evaluated prior to surgery through clinical history in 58 patients. Of the previously potent patients,* de novo* ED occurred in 16.2%. The authors make reference to an impotent patient that recovered erectile function after surgery.

Morey and Mcaninch [56] and Corriere Jr. et al. [57] on the other hand report important rates of recovery of erectile function in impotent patients after posterior urethroplasty. A decrease in impotency rates of 54 to 38% and from 48 to 32%, respectively, was observed. No* de novo* ED was reported in both studies after urethroplasty.

Finally, we can make use of a subanalysis of six studies specifically dealing with posterior urethroplasties, analyzing erectile function before and after urethroplasty, encompassed in a broader meta-analysis, discussed later [58]. After elimination of one of the studies in order to improve heterogeneity and thus improve the statistical quality, The analysis reveals advantage for postsurgical status (24,01% versus 43,27%; OR 2,51; 95% CI: 1,82–3,45; *P* < 0,001).

With the limitations already mentioned, posterior urethroplasty does not seem to play a significant deleterious effect* per se* on erectile function. The overwhelming majority of these patients have ED prior to surgery and surgical reconstruction might even be beneficial in a subgroup of these patients. Aspects such as removal of fibrosis and scar tissue, essential from a technical point of view to achieve a successful urethroplasty, could lead to decompression of nerve structures and allow recovery of function. Restoration of micturition and the simple removal of a suprapubic catheter, allowing improvement of psychological aspects and self-image, may also play a role in this regard.

#### 3.2.5. Meta-Analysis

Two fairly recent meta-analysis sought to systematize the available studies and shed some light on the subject (Table 5) [58–60].

Feng et al. [58] examined 790 studies of which only 23 met the predefined inclusion criteria (randomized controlled trial (RCT) and cohort studies), corresponding to 1729 patients undergoing anterior and posterior urethroplasties. Five of these studies globally analyzed erectile function before and after urethroplasty and found no significant differences between pre- and postoperative scores [OR 0.85; 95% CI (0,52–1,4); *P* = 0,53]. The authors conducted several subanalyses. According to the location of the stenosis, a single study compared the incidence of ED before and after penile urethroplasty specifically and found no statistically significant differences (23.53% versus 35.29%, *P* = 0,45). Only two studies compared penile with bulbar substitution urethroplasties and also found no significant differences in erectile scores (23.81% versus 16.67% OR 1.62; 95% CI: 0.51 to 5.81, *P* = 0,41). These same two studies allowed comparison of buccal mucosa substitution urethroplasties with anastomotic end-to-end urethroplasties revealing lower ED rates for substitution versus anastomotic (OR 0.32; 95% CI: 0.11 to 0.93; *P* = 0,04). Finally only one study analyzed ED occurrence before and after bulbar end-to-end urethroplasty; no statistically significant differences were found (24.14% versus 27.59%; *P* = 0,76). Regarding posterior urethra, the meta-analysis analyzes aspects such presence versus absence of surgical history, immediate repair versus deferred repair, and primary alignment versus immediate repair; all have discarded statistically significant differences.

In turn Blaschko et al. [60] analyzed studies related to the last 15 years in English language, covering only anterior urethroplasties carried out in adulthood, specifically looking for the occurrence of ED as a consequence of urethroplasty. Of the 736 identified articles, 36 met the inclusion criteria, including 2323 patients who were subject to statistical analysis.* De novo* ED incidence varied between 0 and 38%, and generally it was a very rare occurrence, 1% (CI 1–3). The incidence of* de novo* ED did not increase when patients were directly asked about erectile function [OR 0.83 (CI 0.06 to 10.90)] and there was no association with the location or mean stricture length, type of repair, or number of previous failed instrumentation, urethrotomies or urethroplasties. In the overwhelming majority of cases* de novo* ED resolved spontaneously 6–12 months after surgery; 7 of the 21 studies that have registered the occurrence of* de novo* ED also reported occurrence of ED resolution in 86% (50/58) of cases. There was substantial heterogeneity in the studies (*I*-squared = 93%, *P* < 0,001), attributable in part to the variation in how ED was reported.

In summary, the limitations already mentioned obviously make it impossible to provide complete definitive answers regarding the relationship between urethroplasty and erectile dysfunction. Although it is unwise to assume that there is no relationship (in particular when dealing with any individual patient), globally, the evidence accumulated to date, encompassing progressive methodological and statistical quality, seems to point only to a small deleterious role for either anterior or posterior urethroplasty.

### 3.3. Ejaculatory Dysfunction

Table 6 summarizes the studies that address the impact of various types of urethroplasty in ejaculatory function [3, 27, 32, 33, 61–63]. Although most of the studies are retrospective and merely assessed the presence of anterograde ejaculation, most used validated instruments, ejaculatory domains of MSHQ or BMFSI.

Most authors comparing pre- and postoperative scores report significant improvement in ejaculatory function [27, 32, 33]. This result is perfectly understandable, given the considerable improvement in urethral caliber achieved, resulting in better expulsive capacity of the seminal fluid. Authors like Erikson et al. [61] although not finding overall statistically significant differences between pre- and postoperative scores refer to statistically significant improvements in men with ejaculatory dysfunction preoperatively.

Authors as Barbagli et al. [3] identified ejaculatory dysfunction after anastomotic urethroplasty in the form of decreased ejaculatory flow or need for urethral milking surpassing 20%, so that some caution is needed when analyzing these results.

In order to minimize this potential problem, several authors have proposed some minimally invasive procedures in an attempt to maximally preserve structures involved in ejaculatory mechanics. Authors like Barbagli and Kulkarni [23, 24] present approaches referred to as “muscle and nerve sparing urethroplasty” or “one sided urethroplasty,” which aim to minimize the potential iatrogeny over the bulbospongiosus muscle or its innervation. Advantages from these technical refinements remain thus far in the field of theoretical or anatomophysiological hypothesis. Results obtained were not disclosed and as such these approaches lack appropriate validation in this specific context.

### 3.4. Orgasmic Dysfunction

Two studies evaluated this component of sexuality in the context of urethroplasty (Table 7) [28, 35]. In both cases the orgasmic domain was analyzed together with the other domains of IIEF. Both works refer to case series with less than 20 patients on various circumstances, urethroplasty for hypospadias and bulbar urethroplasty. None of the authors found any negative influence of urethroplasty on orgasmic function.

Although a virtual absence of literature on the subject makes it difficult to draw any critical analysis on the subject, a lack of influence of urethral surgery on orgasm is not surprising, since orgasm is essentially considered a neurophysiological phenomenon [64].

### 3.5. Fertility

There are some specific articles that address fertility in the context of urethroplasty (Table 8) [52, 63, 65]. These studies essentially describe the seminal parameters of patients having undergone posterior urethroplasty. There is no reference to cases of azoospermia. Of course it is impossible to implicate the surgical procedure with these findings, although the relationship does not seem obvious. Anyway, due to the high prevalence of male factor infertility in the general population, it is impossible to draw any conclusion.

## 4. Conclusions

Although there are a lot of series describing the results achieved with various types of urethroplasties, the andrological aspects of this pathology and its treatment(s) are clearly insufficiently studied. The available literature is confusing, dispersed, not systematized, and often containing methodological deficits. Although we have been assisting in recent efforts in an attempt to obtain more and better data, there are still obvious gaps that prevent valid conclusions on the subject. Large scale, prospective investigations using standardized validated questionnaires are needed to reliably elucidate the real impact of urethroplasty on the different domains of sexual function.

## Figures and Tables

**Table 1:** Anterior urethra, erectile dysfunction: prospective studies.

Authors, year	*n*	Questionnaire	Follow-up (months)	Age (median)	Results
Sharma et al., 2011 [27]	34	BMSFI	3	34,6	Preop BMSFI: 10,21; postop: 10,34 *P* = 0,554, NSD

Anger et al., 2007 [28]	25	IIEF	6,2	39	Preop IIEF: 62,6; postop: 59,6 *P* = 0,29, NSD

Dogra et al., 2011 [29]	78	IIEF-5	15,5	38,1	38% of postop ED “96% recovery by 6 months,” NSD

Raber et al., 2005 [30]	30	IIEF	51	42	Preop IIEF5: 24 ± 8,7; postop, 24,8 ± 4,96 *P* = 0,77, NSD

Erickson et al., 2010 [31]	52	IIEF	7,2	40,6	Preop IIEF5: 18,7; postop:12,6 *P* < 0,0001: SSD “90% of postop ED recovered by 6 months”

IIEF: International Index of Erectile Function; BMSFI: Brief Male Sexual Function Inventory; NSD: no statistical difference; SSD: statistically significant difference.

**Table 2:** Anterior urethra, erectile dysfunction: retrospective studies.

Authors, year	*n*	Questionnaire	Follow-up (months)	Age (median)	Results
Singh et al., 2010 [32]	150	BMSFI	>3	40	Mean preop BMSFI EF: 9,1; postop: 8,8 *P* = 0,39, NSD

Erickson et al., 2007 [33]	52	BMSFI	22,3	41,7	Mean preop BMSFI EF: 9,2; postop: 8,8 *P* = 0,11, NSD

Coursey et al., 2001 [34]	174	NVQ	36	43,8	69,1% no difference in erectile function 30,9% worsened erectile function

Nelson et al., 2005 [35]	11	IIEF	56,4	30,6	0% ED

Morey and Kizer, 2006 [36]	22	NVQ	26,1	39,95	No difference between end-to-end and extended anastomotic techniques or other types of penile surgery

Welk and Kodama, 2012 [37]	44	NVQ	40	27,6	No difference between nontransecting APA and dorsal graft

Ekerhult et al., 2013 [38]	169	NVQ	12–132	16–75	No difference between anastomotic repair and onlay

IIEF: International Index of Erectile Function; ED: erectile dysfunction; BMSFI: Brief Male Sexual Function Inventory; NSD: no statistical difference; NVQ: nonvalidated questionnaire.

**Table 3:** Anterior and posterior urethra, erectile dysfunction: other prospective studies.

Authors, year	*n*	Study type	Questionnaire	Follow-up (months)	Age (median)	Results
Lumen et al., 2011 [42]	20	P	IIEF-5	6	48	Mean preop IIEF5: 15; postop: 11,62 *P* = 0,11,NSD

Xie et al., 2009 [43]	125	P	IIEF-5 SLQQ	27,3	?	Mean preop IIEF5: 16,57; postop: 17,22 “significant decrease in EEIF-5 at 3 months but not at 6 months”

Johnson and Latini, 2011 [44]	37	P	IIEF-5	9	45	Mean preop IIEF5: 15; postop: 10 *P* = 0,39,NSD

IIEF: International Index of Erectile Function; SLQQ: Sexual Life Quality Questionnaire; ED: erectile dysfunction; BMSFI: Brief Male Sexual Function Inventory; NSD: no statistical difference; P: prospective.

**Table 4:** Posterior urethra, erectile dysfunction.

Authors, year	*n*	Study type	Questionnaire	Follow-up (years)	Age (median)	Results
Anger et al., 2009 [47]	26	R	IIEF	4,4	40,2	54% ED 31% severe ED

Corriere, 2001 [48]	60	R	?	27,3	35	33% “complete” ED

Shenfeld et al., 2003 [49]	25	D	—	<3	28,6	72% preoperative ED

Koraitim, 2005 [10]	155	R	—	1–22	21	34% “definitive” ED 2% after surgery

Mundy, 1996 [9]	82	R	?	>5	?	7% “permanent” ED

Yin et al., 2011 [50]	76	R	—	42,5	34,5	95% remained potent 5% *de novo *ED 59% recovered potency

Lumen et al., 2009 [51]	61	R	—	5,58	34	32,8% ED previous to surgery 2 cases spontaneous recovery

Onen et al., 2005 [52]	49	R	NVQ	12	20	18,4% ED at last follow-up

Mouraviev et al., 2005 [53]	96	R	—	8,8	?	34% after realignment 42% after delayed repair

Tunç et al., 2000 [54]	58	R	—	3,9	24,2	16,2% *de novo *ED

Aboutaieb et al., 2000 [55]	35	R	—	?	25	18,3% ED early repair 5,3% ED delayed repair

Morey and Mcaninch, 1997 [56]	82	R	—	>1	?	54% ED previous to repair 38% ED after repair

Corriere et al., 1994 [57]	50	R	—	>1	?	48% ED previous to repair 32% ED after repair

IIEF: International Index of Erectile Function; NVQ: nonvalidated questionnaire; ED: erectile dysfunction; P: prospective; R: retrospective; D: descriptive.

**Table 5:** Meta-analyses, erectile dysfunction.

Meta-analyses	Studies	*n*	Urethroplasty	Results
Feng et al. [58]	23	1729	Anterior and posterior urethroplasty	No significant difference before or after urethroplasty [OR 0,85; 95% CI (0,52–1,40); *P* = 0,53] ED incidence after posterior urethroplasty lower than before [24,01% versus 43,27%; OR 2,51; 95% CI (1,82–3,45); *P* < 0,01] No significant difference before or after penile urethroplasty [23,53% versus 35,29%; CI (0,13–2,52); *P* = 0,45] No significant difference between penile and bulbar urethroplasty [23,81% versus 16,67% OR 1,62; 95% CI: 0,51–5,81, *P* = 0,41] ED incidence after graft urethroplasty significantly lower than anastomotic urethroplasty [(OR 0,32; 95% CI: 0,11–0,93; *P* = 0,04)]

Blaschko et al. [60]	36	2323	Anterior urethroplasty	1% incidence of *de novo* ED after urethroplasty ED transient and resolved between 6 and 12 months in 86% of cases No statistical significant association between *de novo* ED and stricture location, mean stricture length, number of previous instrumentations/repairs, or type of repair

**Table 6:** Ejaculation.

Authors, year	*n*	Study type	Questionnaire	Follow-up (months)	Urethroplasty	Age (median)	Results
Singh et al., 2010 [32]	150	R	BMSFI	>3	AU	40	Mean preop BMSFI Ej: 4,7; postop: 6,3 *P* < 0,001, improved

Sharma et al., 2011 [27]	34	P	BMSFI	3	AU	34,6	Mean preop BMSFI Ej: 4,68; postop: 6,71 *P* = 0,00, improved

Erickson et al., 2007 [33]	52	R	BMSFI	4	AU	41,7	Mean preop BMSFI Ej: 5,3; postop: 6,2 *P* < 0,04, improved

Barbagli et al., 2008 [3]	60	R	N. Valid.	68	AU^*∗*^	39	23% ejaculatory dysfunction

Erickson et al., 2010 [61]	43	P	MSHQ	6,8	AU	40,4	Mean preop MSHQ Ej: 25,54; postop: 26,94 *P* = 0,17, NSD overall 70% no change 19% improved 11% decreased

El-Assmy et al., 2015 [62]	58	R	MSHQ	61,3	PU	31,6	8,6 ejaculatory dysfunction

Anger et al., 2008 [63]	32	R	N. Valid.	58,8	PU	38,6	100% antegrade ejaculation 15,6% decreased volume

BMSFI: Brief Male Sexual Function Inventory; MSHQ: Male Sexual Health Questionnaire; AU: anterior urethroplasty; ^*∗*^100% end-to-end anastomosis; NSD: no statistical difference.

**Table 7:** Orgasm.

Authors, year	*n*	Study type	Questionnaire	Follow-up (months)	Urethroplasty	Age (median)	Results
Anger et al., 2007 [28]	25	P	IIEF	>3	AU	39	Mean preop IIEF (orgasmic domain): 8,6; postop: 8,3 *P* = 0,28, NSD

Nelson et al., 2005 [35]	11	R	IIEF	56,4	Hypospadias	30,6	No change “All patients experienced orgasm”

IIEF: International Index of Erectile Function; AU: anterior urethroplasty; NSD: no statistical difference.

**Table 8:** Fertility.

Authors, year	*n*	Study type	Follow-up (months)	Urethroplasty	Age (median)	Results
Anger et al., 2008 [63]	13	D	>3	PU	38,6	46% normal (WHO) 53% oligospermia 8% asthenozoospermia 30% oligozoospermia

Iwamoto et al., 1992 [65]	14	D	56,4	PU	?	50% normal (WHO) 21% oligozoospermia 35% asthenozoospermia

Onen et al., 2005 [52]	19	D	144	PU	20	26,3% abnormal semen parameters (WHO)

D: descriptive; R: retrospective; PU: posterior urethroplasty.
